# Children at Risk of Specific Learning Disorder: A Study on Prevalence and Risk Factors

**DOI:** 10.3390/children11070759

**Published:** 2024-06-22

**Authors:** Leyla Bozatlı, Hasan Cem Aykutlu, Açelya Sivrikaya Giray, Tuğçe Ataş, Çisem Özkan, Burcu Güneydaş Yıldırım, Işık Görker

**Affiliations:** 1 Department of Child and Adolescent Psychiatry, Trakya University, Edirne 22000, Turkey; hasancemaykutlu@trakya.edu.tr (H.C.A.); isikgorker@trakya.edu.tr (I.G.); 2 Department of Disability Studies, Faculty of Education, Trakya University, Edirne 22000, Turkey; acelyagiray@trakya.edu.tr; 3 Artvin State Hospital, Artvin 08000, Turkey; tugce.atas@saglik.gov.tr; 4 İnegöl State Hospital, Bursa 16000, Turkey; cisem.ozkan@saglik.gov.tr; 5 Kırklareli Training and Research Hospital, Kırklareli 39000, Turkey; burcu.yildirim16@saglik.gov.tr

**Keywords:** early signs, specific learning disorder, dyslexia, preschooler

## Abstract

Background: Specific learning disorder (SLD) is a neurodevelopmental condition characterised by significant academic difficulties despite normal intelligence and adequate education. The difficulties with reading, writing, and arithmetic may manifest independently or concurrently at different ages. Early symptoms may appear in preschool, including delays in social skills, motor skills, and language development. This study aimed to assess the prevalence of preschool children at risk for SLD and related psychiatric disorders. Method: Data were collected from 515 preschool children in Edirne City, Turkey, using a screening scale for early symptoms of SLD. Socio-demographic information was obtained, and children at risk were invited for a psychiatric evaluation. Results: The mean age of the participants was 72.5 ± 5.6 months. It was determined that 5.7% of the preschool children who participated in the questionnaire were at risk of SLD according to the screening scale scores. Factors such as a father’s low education, the mother smoking during pregnancy, a longer stay in the neonatal intensive care unit, longer screen time, and consanguinity between parents were associated with an increased risk of SLD. Conclusion: This study emphasises the importance of early identification and intervention for SLD and the need to consider associated psychiatric comorbidities. Identifying the risk factors in preschool children may facilitate timely intervention and prevent academic and social difficulties in later years.

## 1. Introduction

Specific learning disorder (SLD) is a neurodevelopmental disorder in which academic skills are significantly below expected levels despite age, intelligence level, and appropriate education, and its aetiology involves the interaction of genetic, epigenetic, and environmental factors. SLD is a condition that impairs one or more aspects of academic functioning, including reading (dyslexia), writing (dysgraphia), and arithmetic (dyscalculia). These types can occur separately or in combination. The incidence and prevalence of SLD vary widely between studies, depending on the sample size, the method, the screening and diagnostic tools used, and the Diagnostic and Statistical Manual of Mental Disorders (DSM-IV/DSM-5) diagnostic criteria used [1]. In DSM-5, it has been reported that the prevalence of SLD in school-aged children from different languages and cultures is between 5–15%, and the rates of reading disorder and dyscalculia are 4–9% and 3–7%, respectively [2]. In a meta-analysis of studies conducted in India, the prevalence of SLD was reported to be 8% [3]. A study conducted in the United States of America (USA) reported a lifetime prevalence of learning disabilities in children of 9.7% [4]. In another study conducted in Brazil, the prevalence was 7.6% [5]. The number of prevalence studies using diagnostic criteria or scales for SLD is small. However, it is accepted that SLD is relatively common and under-recognised [1].

Although the characteristics of SLD may begin to appear in the preschool years or even at a younger age, the diagnostic process for SLD usually begins with the child’s difficulties with reading comprehension or learning, difficulties with writing or written expression, difficulties with number perception/calculation, and when, as a result of these difficulties, the child’s academic performance is below what would be expected for his or her age. In addition to academic difficulties, these children also have difficulties in other areas during the preschool years. Research has demonstrated that early signs of SLD encompass delays in social skill development, challenges in adhering to rules, and difficulties with individual and group work [6]. Additionally, SLD may manifest as motor skill delay, difficulty with ball/balance games requiring hand-eye coordination, limited comprehension of speech, and delays in receptive and expressive language development [7].

In recent years, there has been a growing emphasis among researchers on the early detection of SLD during preschool years. They have focused on recognising the initial symptoms and implementing early intervention strategies [7,8,9]. Studies highlight that if children at risk of learning disabilities are identified during preschool and provided with suitable intervention programs, the likelihood of receiving an SLD diagnosis during their school years is significantly reduced [10,11,12]. The benefits of early intervention in SLD extend beyond the academic realm. Research suggests that an early diagnosis of SLD in children and interventions employed to address the issues related to the learning, socialisation, and emotional development of these children can help to ameliorate the SLD condition and its negative consequences for the child [13].

Although it is possible to identify and intervene in the early stages of SLD, studies have mostly examined the risk factors, and there is not enough research on the prevalence of the characteristics of SLD in the preschool years. Therefore, our objective was to assess the prevalence of preschool children who are at risk of SLD and related psychiatric disorders.

## 2. Materials and Methods

A two-step study was conducted to identify preschool children at risk of SLD. In the initial phase of the study, we conducted a screening process to identify early symptoms of SLD in preschoolers during the spring semester of the 2021–2022 academic year. A study investigating the prevalence of SLD in the same province found it to be 13.6% [14]. As our research will be conducted in the same city centre, the data from this study were used, and the target sample size was set at 610 people with a 95% confidence level and a margin of error of d = 0.027. To account for stratification by sex, the target number was increased to 671. Following the approval from the Ethics Committee of the Medical Faculty of Trakya University (protocol number 2022/43) and the acquisition of the necessary permissions from the Provincial Directorate of National Education, a stratified random sampling method was employed to select participants from the student class lists of 27 preschools in the province. During this educational period, the city of Edirne had 31 schools with preschool classes, with a total student enrollment of 2022. However, our study was conducted with 27 schools, excluding 3 schools located outside Edirne’s city centre and 1 “special education” school. Thus, the study population comprised students enrolled in these 27 schools. Prior to the commencement of this study, informed written consent was obtained from the parents of the identified students. Children with a known psychiatric disorder, such as autism or cognitive developmental delay, or children whose parents did not provide consent were excluded from the study. The families who consented to participate completed a socio-demographic questionnaire designed by the researchers and a standardised screening tool for early symptoms of SLD.

Students who scored above the established cutoff for risk were invited to undergo a comprehensive psychiatric evaluation at the Trakya University Faculty of Medicine Child Psychiatry Clinic between eight and twelve months later in the subsequent academic year, following the completion of at least six months of the first grade. In addition to assessing SLD, the comprehensive psychiatric evaluation also assessed other psychiatric diagnoses that may be comorbid with SLD, using the Kiddie Schedule for Affective Disorders and Schizophrenia, Current and Lifetime Version [15,16].

### 2.1. Assessment Tools

We used the Learning Disability Early Symptoms Screening Scale [17] to evaluate the early symptoms of specific learning disabilities in preschool children aged 4–6 years. The scale has four subscales with 52 items in total: language development and communication (14 items), cognitive skills (19 items), psychomotor skills (13 items), and social–emotional skills (7 items). The items measure various skills and abilities that may be related to learning disabilities, such as language, memory, attention, motor, and social skills. The items are rated on a 5-point Likert scale, ranging from 1 (strongly disagree) to 5 (strongly agree), with higher scores indicating a higher risk of learning disability. A few examples of some of the questions in the scale can be found in the Supplementary Materials. The scale has a minimum score of 52 and a maximum score of 260. The raw scores of each subscale and the total scale were converted into Z and T scores based on the standardised group mean and standard deviation. The standardised group arithmetic means and standard deviations were obtained for each subtest and the total scale score. The score ranges were calculated for each subtest according to the standard score ranges resulting from the group arithmetic mean and standard deviation obtained, and 4 risk groups were defined according to the score ranges of very low, mild, moderate, and high risk. This provided a detailed screening of the participants for risk categorisation.

To obtain a representative sample of children at risk for SLD, we operationally defined the ‘at-risk’ group as children who scored at a ‘moderate risk’ or ‘high risk’ level on the total scale score or any of the four subscales. Conversely, we categorised the ‘low-risk group’ as participants who obtained scores that were indicative of a ‘very low risk’ or ‘mild risk’.

Kiddie-Schedule for Affective Disorders and Schizophrenia—Present and Lifetime Version is a semi-structured diagnostic interview tool that probes the current and past stages of childhood and adolescent psychiatric disorders [15]. The Turkish version of K-SADS-PL was reported to show good test–retest and interrater reliability [16]. In this study, it was used to diagnose psychiatric disorders accompanying SLD.

### 2.2. Statistical Analysis

In this descriptive study, we computed the statistical measures, including the mean/median, standard deviation/interquartile range, and 95% confidence interval. The prevalence value was also presented as a percentage. To draw comparisons between the quantitative data of children with and without a SLD risk, we employed either the Student’s *t*-test or the Mann–Whitney U test, contingent on the data’s distribution characteristics. The Chi-square test was utilised for the comparison of categorical data. Logistic regression analysis was conducted to evaluate the potential risk factors influencing SLD. The inclusion criteria of the variables in the binary logistic regression model were set among those that were significant (*p* < 0.10) by univariate comparisons. A *p*-value of less than 0.05 was considered statistically significant. All statistical analyses were performed using SPSS version 20 for Windows (IBM SPSS Statistics for Windows, Version 20.0. Armonk, NY, USA: IBM Corp.)

## 3. Results

**Figure 1:**
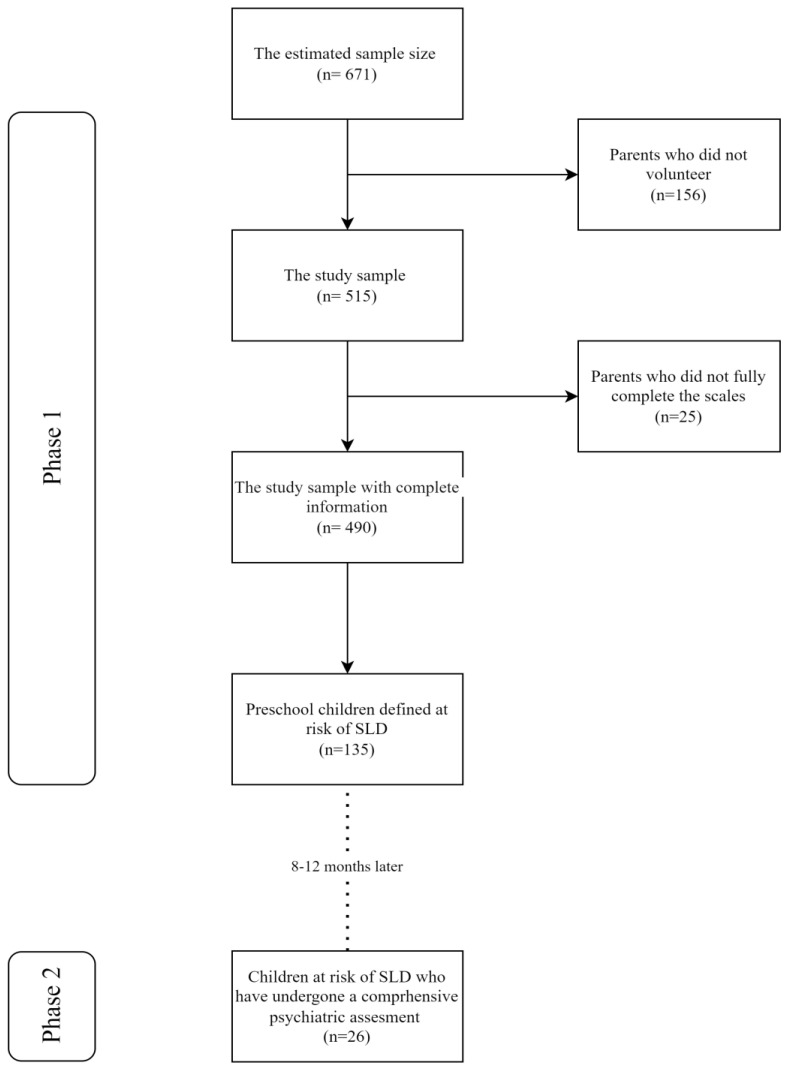
Flowchart of the study sample.

Our primary objective was to conduct a survey involving 671 students. However, due to incomplete responses on the measurement scales or a lack of willingness to participate, we were only able to analyse data from 490 students, as provided by their parents (Figure 1). The mothers completed 89.9% of the forms. The mean age of the participants was 72.5 ± 5.6 SD months, and 50.7% of the children were female (Table 1).

**Figure 2:**
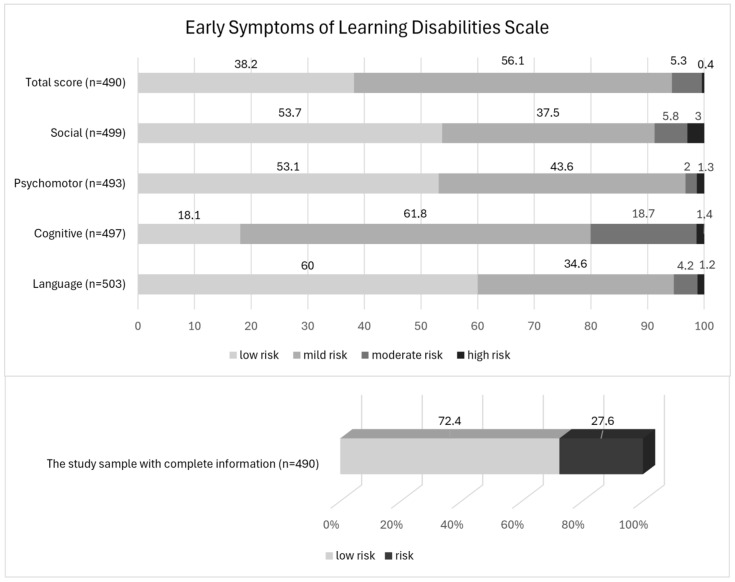
The specific learning disorder risk groups.

A total of 27.6% (n = 135) of the study sample was identified as at risk for SLD (Figure 2). The SLD risk group had statistically significantly higher scores in four subscales of the Early Symptoms of Learning Disabilities Screening Scale (language development and communication skills, cognitive skills, psychomotor skills, psychosocial skills, and social–emotional skills) compared to the low-risk group (Table 2).

We compared the socio-demographic data between the groups and found that fathers of children in the high-risk group had a lower level of education (*p* = 0.017), mothers smoked more during pregnancy (*p* = 0.004), and children stayed longer in the neonatal intensive care unit (*p* = 0.039), had more daily screen time (*p* = 0.009), and had more consanguineous parents (*p* = 0.023) (Table 3).

Multiple regression analysis showed that consanguinity between parents (*p* = 0.014, Exp(B) = 6) and more screen time (*p* = 0.033, Exp(B) = 1.002) also predicted the risk group. The logistic regression model was statistically significant (X2(10) = 20.058 and *p* = 0.029) and well-fitted to the data, as indicated by the correlations of estimates and the Hosmer–Lemeshow goodness-of-fit test (X2(8) = 3.535, *p* = 0.896). The model explained 69% of the variance in SLD, as measured by Nagelkerke R2 (Table 4).

In the study’s second phase, children at risk for SLD were invited to attend the child psychiatry outpatient clinic for clinical assessment. Nevertheless, only 19.3% (n = 26) of the children and their families visited the clinic, allowing for their assessment. Psychiatric disorders were diagnosed in 92.3% (n = 24) of the assessed children. Among the cases with a psychiatric diagnosis, there were also cases with psychiatric comorbidities. The specific psychiatric diagnoses were as follows: 41.7% had SLD (n = 10), 83.3% had ADHD (n = 20), 20.8% had anxiety disorder (n = 5), 8.3% had speech disorder (n = 2), 8.3% had eating disorder (n = 2), 4.2% had OCD (n = 1), and 4.2% had an intellectual disability (n = 1).

While all children identified in the risk group were invited for psychiatric evaluation, only 19.3% of them were examined. To understand the reasons behind the non-attendance of certain families, we compared the characteristics of those who attended the psychiatric evaluation and those who did not. The comparison was based on the following characteristics: gender (*p* = 0.768), presence of siblings (*p* = 0.169), marital status (*p* = 0.482), mother’s employment status (*p* = 0.984), mother’s health problem (*p* = 1.000), maternal education (*p* = 0.365), paternal employment status (*p* = 0.485), paternal health problem (*p* = 0.737), paternal education (*p* = 0.054), kinship (*p* = 0.345), family income (*p* = 0.660), and social security status (*p* = 1.000). No statistically significant differences were found between the two groups based on these characteristics.

## 4. Discussion

We aimed to estimate the prevalence of preschool children at risk of SLD and to follow them up in primary school to see if they received a clinical diagnosis of SLD. Our sample’s total scale score indicated that 5.7% of preschool children were at risk of SLD. When we also examined the subscale scores, the prevalence of children at risk for SLD increased to 27.6%. To our knowledge, no other study has explored the prevalence of children at risk of SLD in this population.

The prevalence of specific learning disorders (SLD) varies across studies from different countries [1]. Epidemiological studies and government reports in 2004–2005 indicated that the prevalence of learning disabilities in Greece was between 1.2% and 1.4%. In contrast, the prevalence rate of SLD among children aged 3–17 years was 9.7% in the United States [4]. Moreover, the prevalence of SLD in primary school children was 7.7% in Pakistan [18] and 13.6% in Turkey [14]. According to the DSM, the prevalence of SLD in reading, writing, and mathematics ranged from 5% to 15% among school-aged children in different languages and cultures [2]. However, some studies have found a higher prevalence of SLD. For example, in a study conducted in India, the prevalence of SLD was 30.77% [19], while in a review article analysing studies conducted in India, this rate was between 2% and 33% [20]. Similarly, in a study in Turkey where symptoms of SLD were screened through a questionnaire form, this rate was reported as 36.8% on teacher forms and 37.9% on parent forms [21]. As a result of the studies and meta-analyses, the reported rates for the prevalence of SLD spanned a very wide range. This wide range is explained by the population and country in which the studies were conducted, the language used, the presence and variety of screening tools in the country, the study method (screening tools, clinical assessment), and whether the study was conducted according to the DSM-IV or DSM-5 criteria before or after 2013 [1]. In our study, the prevalence rate of SLD symptoms was close to the SLD rate reported in the DSM and the literature. Therefore, our SLD risk ratio results (5.7% with the total scale scoring and 27.6% when considering the subscale scores and medium risk group) are consistent with the wide range of SLD prevalence reported in the literature.

When assessing for SLD risk in preschool children, it is important not to overlook other psychiatric disorders that share common symptoms and causes with SLD. In this study, we invited children at risk for specific learning disabilities to undergo a comprehensive psychiatric assessment at our outpatient clinic after a one-year follow-up upon finishing first grade. However, only 19.3% of the children completed the assessment, which was a significantly lower rate than expected. To understand the reason for the low participation, the characteristics of the families who attended the clinical assessment and those who did not (child’s gender, siblings, marital status, parents’ education, employment status, health, family income, social security, and kinship) were analysed, but no difference was found. Although the reason for the low participation is unknown, it suggests that parents’ awareness of SLD may be low. Furthermore, parents may seek assessments for their children who exhibit pronounced behavioural symptoms or developmental delays relative to their age-matched peers.

The psychiatric assessment results showed that 92.3% of the children evaluated had a psychiatric disorder such as attention deficit hyperactivity disorder (ADHD), SLD, anxiety disorder, obsessive-compulsive disorder (OCD), intellectual disability, or an eating disorder. Notably, SLD was diagnosed in 38.46% of these cases. Interestingly, ADHD was diagnosed in 83.3% of the patients, indicating a strong correlation between ADHD and SLD. This association is supported by previous studies, such as the research by Visser et al. (2020), which found that 28% of 3014 children with SLD also had ADHD [22]. Moreover, studies focusing on hyperactive children frequently report academic failure and learning difficulties. Numerous epidemiological studies have also found associations between SLD and hyperactivity, as well as between specific subtypes of ADHD and SLD and their respective symptoms [23]. These findings emphasise the importance of early identification and intervention for children who are at risk of SLD. They also suggest that SLD is a heterogeneous disorder that can manifest in various ways and interact with other disorders. Further research is necessary to investigate the causes, mechanisms, and outcomes of SLD and its comorbidities during the preschool period.

The prevalence of smoking during pregnancy was higher among mothers of children in the risk group for SLD (*p* = 0.004). Studies investigating the effects of maternal smoking have reported an increased risk of preterm birth, low birth weight, ADHD, and learning difficulties [24]. Research has shown that smoking during pregnancy is linked to ADHD in the child [25,26]. Additionally, mothers who smoke more than 20 cigarettes per day during pregnancy have an increased risk of their children developing intellectual disability [27]. Furthermore, smoking during pregnancy has long-term negative effects. According to Fergusson et al. (1998), smoking during pregnancy is associated with an increase in psychiatric symptoms in late adolescence. The study also reported that exposure to cigarette smoke during pregnancy is associated with increased rates of psychiatric symptoms with increasing doses. In conclusion, studies have shown that smoking during pregnancy is associated with early and late psychiatric symptoms and diagnoses [28]. Our study found that the SLD risk group was diagnosed with ADHD (83.3%), anxiety disorder (20.8%), an eating disorder (8.3%), a speech disorder (8.3%), OCD (4.2%), and intellectual disability (4.2%) in addition to SLD. This evidence further corroborates the existing body of research in this field.

Our study revealed that fathers of children at risk for SLD had lower educational levels (*p* = 0.017). This finding aligns with a previous study conducted in the same region, which associated lower paternal education with an increased risk of SLD in children [14]. In the broader context, research on early childhood development often considers maternal or parental education as a whole [29,30]. However, few studies have separately examined the impact of the father’s educational level [31]. One such study, conducted across 44 low- and middle-income countries, analysed the relationship between both maternal and paternal education levels and children’s early development. The results indicated a correlation between the parents’ educational level and early childhood development. Specifically, higher maternal and paternal education levels were linked to better early childhood development outcomes. Furthermore, the parents’ educational levels were positively associated not only with their own parenting practices but also with the other parents’ interactions with the child. The study also found that the manner in which caregivers support the child’s learning process influences the child’s development significantly [31].

Intensive care in the neonatal period can have long-term consequences for a baby’s health. However, these situations are complex and depend on many factors. Babies may need intensive care for a variety of reasons, including prematurity, complications during labour, infections, or other health problems. On the other hand, intensive care can disrupt the natural bonding process essential for children’s growth and development. In our study, the rate of neonatal intensive care stay was higher in cases identified as being at risk of SLD (*p* = 0.039). Similarly, many researchers have reported that children treated in the intensive care unit have significant educational problems [32,33].

We found that children born from consanguineous marriages had a higher likelihood of being in the SLD risk group (*p* = 0.023). When we applied multiple regression to the data, consanguineous marriage was one of the two risk factors that remained significant for SLD risk (*p* = 0.014, Exp(B) = 6). Consanguineous marriage is defined as a marriage between close biological relatives (first and second cousins). The rate of consanguineous marriages in different countries depends on various factors such as education level, religion, local traditions, and socio-economic status [34]. Consanguineous marriages, especially between first cousins, are associated with an increased incidence of several inherited diseases, including intellectual disability and psychiatric disorders [35,36]. Studies have suggested possible negative consequences of consanguineous marriage, such as reduced fertility and an increased risk of infant mortality, congenital disease, and intellectual disability [37]. There is also considerable evidence that reading disability may be inherited as a family trait. A study investigating reading disability in children born into consanguineous marriages reported a significant association between consanguineous marriage and an increased risk of reading disability, highlighting the effect of genetics. The study assessed the reading skills of 770 students and compared the performance of two experimental groups with reading difficulties: 22 students from first-cousin marriages and 21 children from unrelated parents. The control group consisted of 21 children with typical reading skills. The results showed that children of cousin parents were more likely to have reading difficulties than children of parents from other families [38].

In the current technological era, screens have become an increasingly common aspect of daily life. The impact of these devices (smartphones, tablets, computers, and televisions) that make our lives easier on children’s development and education is of great interest to researchers. Although the use of digital devices is spreading rapidly among very young children, the effects of screen time on emotional and cognitive functions are still being debated. A longitudinal study (4–8 years) assessing emotional regulation and academic performance reported that children’s screen time was positively and significantly associated with mood dysregulation at age 4 and negatively associated with mathematics and literacy performance at age 8 [39]. In a further study that focused on the preschool period, researchers noted that a child’s first screen exposure to television may be formative because of developmental differences between preschool and later childhood. The importance of this period is explained by the fact that habit formation and overexposure in the early period increase the likelihood of overexposure later in life and that screen use tends to increase over time to include more entertainment as opposed to viewing for educational purposes only [40]. Screen time has also been linked to attention span. A meta-analysis of studies examining this relationship reported a negative association between increased screen time and attention span [41]. On the other hand, time spent with parents is also important. In a study that examined the time children spent with four digital media devices (TV, computer, smartphone, and tablet computer) using a nationally representative sample of more than 2300 parents of children aged 0–8 years, results from linear regression analyses showed that parents’ own screen time was strongly related to their children’s screen time. Further analysis shows that children’s screen time use is the result of an interaction between child and parent factors and is strongly influenced by parental attitudes [42]. Due to the role of parents in the development of their children’s physical activity and sedentary behaviour, especially in the early years, the effect of parental influence on screen time and physical activity in young children has been investigated. The results suggest that parental encouragement and support can increase children’s physical activity and that reducing parents’ own screen time can lead to a reduction in children’s screen time. In light of these findings, it has been reported that improving parental self-efficacy or changing parenting styles may be beneficial in increasing young children’s physical activity and reducing screen time [43]. Although it is noted that screens can have a positive effect on accessing information outside the daily routine, learning vocabulary/language, or general cultural information, the key point here is that screen time and content should be under parental control [40].

The significant increase in screen time among the group at risk for SLD (*p* = 0.009), which remained significant after regression analysis (*p* = 0.033), coupled with a high rate of psychiatric diagnosis (92.4%) among those assessed, underscores the need for comprehensive studies on the effects of screen use. This is particularly relevant given the ubiquity of digital media tools in various areas such as education, work, communication, and entertainment. The recent surge in mental health issues among young people has been hypothesised to be linked to increased screen-based technology usage. However, these hypotheses are primarily based on cross-sectional studies. Therefore, more longitudinal studies examining screen content and the motivations for screen use are warranted to elucidate the relationship between screen time and mental health symptoms [44]. Concurrently, it is crucial to conduct studies on rational digital use and disseminate the findings to society, given the mobile and easily accessible nature of digital media tools.

## 5. Conclusions

Although the diagnostic process for SLD typically commences after primary school, early symptoms can be observed during the preschool years. Our study found a high prevalence of preschool children at risk for SLD, many of whom also have a wide range of psychiatric comorbidities. However, parents are often oblivious to the symptoms. It has been reported that there is a four-year gap between the time a mother first suspects SLD symptoms and the child’s actual diagnosis [45]. This gap can be reduced by identifying the risk factors and clinical signs of SLD in preschool children, as well as by increasing the awareness and involvement of teachers in the referral process. Teachers can facilitate early intervention by recognising the learning difficulties and preferences of each child and by collaborating with child and adolescent psychiatrists to design individualised educational programmes. Further research is necessary to enhance understanding of the risk of SLD and its management.

## Figures and Tables

**Table 1:** Descriptive information of the study sample.

Age	*Month*	72.5 ± 5.6
Sex	*Boy*	49.3% (n = 254)
	*Girl*	50.7% (n = 261)
Parents who filled out the form	*Mother*	88.9% (n = 458)
	*Father*	11.1% (n = 57)
Siblings	*Yes*	69.3% (n = 357)
	*No*	30.7% (n = 158)
	*Number of siblings*	1 (1–3)
	*First-born*	53.2% (n = 274)
	*Second-born*	41% (n = 211)
	*Third-born*	5.2% (n = 27)
	*Fourth-born*	0.6% (n = 3)
Marital Status	*Together*	93.2% (n = 480)
	*Divorced*	3.5% (n = 18)
	*Separated*	1% (n = 5)
	*Death*	0.4% (n = 2)
	*Missing value*	1.9% (n = 10)
Number of people living in the home		4 (2–7)
Mother’s age		35.6 ± 5.1
Mother’s job	*Working*	40% (n = 298)
	*Not working*	57.9% (n = 298)
	*Missing value*	2.1% (n = 11)
Mother’s education	*Illiterate*	0.4% (n = 2)
	*Literate*	0.6% (n = 3)
	*Primary school*	6% (n = 31)
	*Secondary school*	12% (n = 62)
	*High school*	36.7% (n = 189)
	*University*	42.5% (n = 219)
	*Missing value*	1.7% (n = 9)
Father’s age		38.77 ± 5.41
Father’s job	*Working*	94.4% (n = 486)
	*Not working*	3.5% (n = 18)
	*Missing value*	2.1% (n = 11)
Father’s education	*Illiterate*	0.2% (n = 1)
	*Primary school*	5.4% (n = 28)
	*Secondary school*	15.1% (n = 78)
	*High school*	38.4% (n = 198)
	*University*	38.7% (n = 199)
	*Missing value*	2.1% (n = 11)
Family income	*Less than 5000 TL*	21.7 (n = 112)
	*5–10,000 TL*	41.9% (n = 216)
	*10–15,000 TL*	21.6% (n = 111)
	*More than 15,000 TL*	10.5% (n = 54)
	*Missing value*	4.3% (n = 22)

n (%), mean ± SD, median (min–max).

**Table 2:** Early symptoms of learning disabilities screening scale scores between the risk and low-risk groups.

	Low-Risk Group Median (Min–Max) (n = 355)	Risk Group Median (Min–Max) (n = 135)	*p* ^a^
Language Development and Communication Skills	3 (1–9)	6 (1–20)	<0.001
Cognitive Skills	6 (1–9)	10 (1–19)	<0.001
Psychomotor Skills	3 (1–9)	6 (1–20)	<0.001
Social–Emotional Skills	3 (1–19)	7 (1–19)	<0.001
Total Score	87 (4–131)	120 (20–247)	<0.001

^a^: Mann–Whitney U test.

**Table 3:** Comparison of the specific learning disorder risk groups for variables.

		Low-Risk Group (n = 355)	Risk Group (n = 135)	*p*
Age	*Month*	72 (IQR = 6)	71 (IQR = 7)	0.510 ^MU^
Sex	*Boy*	48.2% (n = 171)	52.6% (n = 71)	0.382 ^CS^
	*Girl*	51.8% (n = 184)	47.4% (n = 64)	
Siblings	*Yes*	68.5% (n = 243)	69.6% (n = 94)	0.801 ^CS^
	*No*	31.5% (n = 112)	30.4% (n = 41)	
Number of siblings		1 (IQR = 0)	1 (IQR = 0)	0.728 ^MU^
Marital Status	*Together*	94% (n = 330)	97.7% (n = 130)	0.325 ^CS^
	*Divorced*	4.3% (n = 15)	2.3% (n = 3)	
	*Separated*	1.4% (n = 5)	0%	
	*Parental loss*	0.3% (n = 1)	0%	
Number of people living in the home		4 (IQR = 1)	4 (IQR = 1)	0.685 ^MU^
Mother’s age		35 (IQR = 7)	35 (IQR = 7)	0.370 ^MU^
Mother’s job	*Working*	42.9% (n = 150)	38.6% (n = 51)	0.402 ^CS^
	*Not working*	57.1% (n = 200)	61.4% (n = 81)	
Mother’s education	*Low*	6.3% (n = 22)	9.8% (n = 13)	0.216 ^CS^
	*Medium*	47.4% (n = 167)	51.1% (n = 68)	
	*High*	46.3% (n = 163)	39.1% (n = 52)	
Father’s age		38 (IQR = 7)	38 (IQR = 7)	0.708 ^MU^
Father’s job	*Working*	96% (n = 336)	97.7% (n = 129)	0.580 ^f^
	*Not working*	4% (n = 14)	2.3% (n = 3)	
Father’s education	*Low*	3.4% (n = 12)	9.8% (n = 13)	0.017 ^CS^
	*Medium*	54.9% (n = 192)	53.4% (n = 71)	
	*High*	41.7% (n = 146)	36.8% (n = 49)	
Family income	*Low*	19.6% (n = 67)	29% (n = 38)	0.087 ^CS^
	*Medium*	69.6% (n = 238)	61.1% (n = 80)	
	*High*	10.8% (n = 37)	9.9% (n = 13)	
Planned pregnancy	*yes*	81.4% (n = 289)	79.3% (n = 107)	0.589 ^CS^
	*no*	18.6% (n = 66)	20.7% (n = 28)	
Assisted reproductive techniques	*yes*	7.4% (n = 26)	5.9% (n = 8)	0.566 ^CS^
	*no*	92.6% (n = 325)	94.1% (n = 127)	
Threat of miscarriage	*yes*	15.3% (n = 54)	16.4% (n = 22)	0.761 ^CS^
	*no*	84.7% (n = 299)	83.6% (n = 112)	
Medical illness in mother during pregnancy	*yes*	8.2% (n = 29)	9.6% (n = 13)	0.618 ^CS^
	*no*	91.8% (n = 324)	90.4% (n = 122)	
Medication use in pregnancy	*yes*	28% (n = 98)	26.1% (n = 35)	0.678 ^CS^
	*no*	72% (n = 252)	73.9% (n = 99)	
Smoking before pregnancy	*yes*	29.6% (n = 105)	37.8% (n = 51)	0.082 ^CS^
	*no*	70.4% (n = 250)	62.2% (n = 84)	
Smoking during pregnancy	*yes*	5.6% (n = 20)	13.3% (n = 18)	0.004 ^CS^
	*no*	94.4% (n = 335)	86.7% (n = 117)	
Alcohol use during pregnancy	*yes*	0%	0.8% (n = 1)	0.274 ^f^
	*no*	100% (n = 352)	99.2% (n = 132)	
Supplementary use in pregnancy	*yes*	86.7% (n = 301)	90.8% (n = 119)	0.221 ^CS^
	*no*	13.3% (n = 46)	9.2% (n = 12)	
Gestation	*week*	38 (IQR = 1)	38 (IQR = 1.3)	0.627 ^MU^
Birth length	*cm*	50 (IQR = 4)	50 (IQR = 3)	0.496 ^MU^
Birth weight	*gr*	3300 (IQR = 680)	3350 (IQR = 742.5)	0.620 ^MU^
Postpartum intensive care unit hospitalisation	*yes*	9.1% (n = 32)	15.6% (n = 21)	0.039 ^CS^
	*no*	90.9% (n = 321)	84.4% (n = 114)	
Breastfeeding	*month*	18 (IQR = 18)	18 (IQR = 18)	0.858 ^MU^
Walking milestone	*month*	12 (IQR = 2)	12 (IQR = 2.3)	0.058 ^MU^
Sentence formation milestone	*month*	18 (IQR = 11.8)	18 (IQR = 12)	0.860 ^MU^
History of epilepsy	*yes*	2% (n = 7)	3% (n = 4)	0.506 ^CS^
	*no*	98% (n = 346)	97% (n = 131)	
History of surgery	*yes*	10.2% (n = 36)	11.9% (n = 16)	0.585 ^CS^
	*no*	89.8% (n = 316)	88.1% (n = 118)	
History of head trauma	*yes*	0.3% (n = 1)	1.5% (n = 2)	0.187 ^f^
	*no*	99.7% (n = 351)	98.5% (n = 133)	
Hand preference	*right*	84.7% (n = 300)	88.1% (n = 119)	0.565 ^CS^
	*left*	10.5% (n = 37)	8.9% (n = 12)	
	*both*	4.8% (n = 17)	3% (n = 4)	
Screen time	*minute*	180 (IQR = 120)	210 (IQR = 150)	0.009 ^MU^
Mother’s reading milestone	*1st class with peers*	98.3% (n = 343)	96.1% (n = 124)	0.555 ^CS^
	*1st class later than peers*	0.9% (n = 3)	1.6% (n = 2)	
	*2nd class*	0.6% (n = 2)	1.6% (n = 2)	
	*3rd class or later*	0.3% (n = 1)	0.8% (n = 1)	
Father’s reading milestone	*1st class with peers*	98% (n = 342)	97.7% (n = 128)	0.750 ^CS^
	*1st class later than peers*	0.9% (n = 3)	1.5% (n = 2)	
	*2nd class*	0.6% (n = 2)	0.8% (n = 1)	
	*3rd class or later*	0.6% (n = 2)	0%	
Kinship	*Yes*	1.4% (n = 5)	5.3% (n = 7)	0.023 ^f^
	*No*	98.6% (n = 346)	94.7% (n = 126)	
Degree of kinship	*1st*	20% (n = 1)	16.7% (n = 1)	0.946 ^CS^
	*2nd*	40% (n = 2)	33.3% (n = 2)	
	*3rd*	40% (n = 2)	50% (n = 3)	

^MU^ Mann–Whitney U test, ^CS^ Pearson Chi-square, ^f^ Fischer’s exact test, *p* < 0.05.

**Table 4:** Regression analysis for predicting SLD risk.

		B	S.E.	Wald	df	Sig.	Exp(B)	95% I. for Exp(B)	
								Lower	Lower
Father’s education	Low			3.015	2	0.222			
	Medium	−0.887	0.512	3.001	1	0.083	0.412	0.151	1.124
	High	−0.793	0.536	2188	1	0.139	0.452	0.158	1.294
Family income	Low			0.951	2	0.622			
	Medium	−0.230	0.294	0.614	1	0.433	0.794	0.447	1.413
	High	0.033	0.449	0.005	1	0.941	1.034	0.429	2.493
Smoking before pregnancy (yes)		0.114	0.272	0.176	1	0.674	1.121	0.658	1.909
Smoking during pregnancy (yes)		0.191	0.456	0.176	1	0.675	1.211	0.496	2.958
Postpartum intensive care unit hospitalistion (yes)		0.375	0.343	1.192	1	0.275	1.454	0.742	2.850
Walking milestone		0.004	0.042	0.008	1	0.929	1.004	0.925	1.090
Screentime		0.002	0.001	4.527	1	0.033	1.002	1.000	1.004
Kinship (yes)		1.792	0.729	6.040	1	0.014	6.000	1.437	25.043
Constant		−0.683	0.783	0.760	1	0.383	0.505		

## Data Availability

The data presented in this study are available on request from the corresponding author due to privacy restriction.

## Supplementary Materials

The following supporting information can be downloaded at: https://www.mdpi.com/article/10.3390/children11070759/s1, The Learning Disability Early Symptoms Screening Scale (LDESSS).
