# Supplementary material for: Children at Risk of Specific Learning Disorder: A Study on Prevalence and Risk Factors
Source: Children (Basel). 2024 Jun 22;11(7):759. doi: 10.3390/children11070759 (PMC11274916; doi:10.3390/children11070759)
Supplement: Supplementary file 1 [file children-11-00759-s001.zip › children-3049870-supplementary.pdf]

The Learning Disability Early Symptoms Screening Scale (LDESSS)

Examples of scale questions: (The question items were randomly selected by the authors and translated from Turkish to English. In the original version of the questionnaire, additional explanations were included for the understanding of the items by the families.)

<u>Language development and communication skills related items-14 items</u>

2. My child has problems saying words correctly.

5. My child has difficulty transferring what he/she has heard to another person.

8. My child has difficulty choosing appropriate words when speaking.

9. The variety of words my child uses in speech is limited compared to his/her peers.

13. My child has difficulty naming familiar objects.

<u>Cognitive skills related items - 18 items</u>

3. My child needs reminders about what he/she should do in the tasks assigned to him/her.

7. My child acts without thinking and does the first thing that comes to mind.

9. My child wants a promise to be fulfilled immediately.

11. My child has difficulty in maintaining group play with peers.

17. My child often needs help/guidance when trying

<u>Psychomotor skills related items- 13 itemsnew activities.</u>

2. My child wears slippers or shoes backwards.

5. My child has difficulty cutting paper etc. with scissors.

8. My child spills food on him/herself when eating.

13. My child has difficulty drawing or copying.

<u>Social-emotional skills related items - 7 items</u>

2. My child has difficulty adapting to new or unfamiliar environments.

6. My child hesitates/shies away from making a request from someone.

7. My child makes preferences for games and toys at the level of younger children.
